# Threonine 89 Is an Important Residue of Profilin-1 That Is Phosphorylatable by Protein Kinase A

**DOI:** 10.1371/journal.pone.0156313

**Published:** 2016-05-26

**Authors:** David Gau, William Veon, Xuemei Zeng, Nathan Yates, Sanjeev G. Shroff, David R. Koes, Partha Roy

**Affiliations:** 1 Department of Bioengineering, University of Pittsburgh, Pittsburgh, Pennsylvania, United States of America; 2 Biomedical Mass Spectrometry Center, University of Pittsburgh, Pittsburgh, Pennsylvania, United States of America; 3 Department of Cell Biology, University of Pittsburgh, Pittsburgh, Pennsylvania, United States of America; 4 Department of Computational and Systems Biology, University of Pittsburgh, Pittsburgh, Pennsylvania, United States of America; 5 Department of Pathology, University of Pittsburgh, Pittsburgh, Pennsylvania, United States of America; McGill University Department of Neurology and Neurosurgery, CANADA

## Abstract

**Objective:**

Dynamic regulation of actin cytoskeleton is at the heart of all actin-based cellular events. In this study, we sought to identify novel post-translational modifications of Profilin-1 (Pfn1), an important regulator of actin polymerization in cells.

**Methodology:**

We performed *in vitro* protein kinase assay followed by mass-spectrometry to identify Protein Kinase A (PKA) phosphorylation sites of Pfn1. By two-dimensional gel electrophoresis (2D-GE) analysis, we further examined the changes in the isoelectric profile of ectopically expressed Pfn1 in HEK-293 cells in response to forskolin (FSK), an activator of cAMP/PKA pathway. Finally, we combined molecular dynamics simulations (MDS), GST pull-down assay and F-actin analyses of mammalian cells expressing site-specific phosphomimetic variants of Pfn1 to predict the potential consequences of phosphorylation of Pfn1.

**Results and Significance:**

We identified several PKA phosphorylation sites of Pfn1 including Threonine 89 (T89), a novel site. Consistent with PKA’s ability to phosphorylate Pfn1 *in vitro*, FSK stimulation increased the pool of the most negatively charged form of Pfn1 in HEK-293 cells which can be attenuated by PKA inhibitor H89. MDS predicted that T89 phosphorylation destabilizes an intramolecular interaction of Pfn1, potentially increasing its affinity for actin. The T89D phosphomimetic mutation of Pfn1 elicits several changes that are hallmarks of proteins folded into alternative three-dimensional conformations including detergent insolubility, protein aggregation and accelerated proteolysis, suggesting that T89 is a structurally important residue of Pfn1. Expression of T89D-Pfn1 induces actin:T89D-Pfn1 co-clusters and dramatically reduces overall actin polymerization in cells, indicating an actin-sequestering action of T89D-Pfn1. Finally, rendering T89 non-phosphorylatable causes a positive charge shift in the isoelectric profile of Pfn1 in a 2D gel electrophoresis analysis of cell extracts, a finding that is consistent with phosphorylation of a certain pool of intracellular Pfn1 on the T89 residue. In summary, we propose that T89 phosphorylation could have major functional consequences on Pfn1. This study paves the way for further investigation of the potential role of Pfn1 phosphorylation in PKA-mediated regulation of actin-dependent biological processes.

## Introduction

Actin-based cell migration is fundamental to a number of physiological processes including, but not limited to, embryonic development, wound healing and inflammatory response. Cell migration correlates with marked changes in the actin cytoskeleton where a motile cell has extremely rapid and directed actin polymerization and turnover [1]. Membrane protrusion of the leading edge is the initiating event of cell migration and requires *de novo* actin nucleation followed by filament elongation and/or elongation of pre-existing filaments, catalyzed by various nucleation-promoting and elongating factors (e.g. N-WASP/WAVE, formins and Ena/VASP). These factors harbor poly-L-proline (PLP) domains through which they interact with profilins (Pfn), a family of actin-monomer binding proteins that strongly inhibits spontaneous nucleation and elongation at the pointed ends of actin filaments but promotes barbed-end elongation through the addition of ATP-bound monomeric actin [2]. Interaction with Pfn1 (the most abundant isoform of Pfn in mammals) enhances the actin polymerizing abilities of nucleation-promoting and elongating factors *in vitro* and *in vivo* [3–6].

How Pfn1’s interactions with its ligands are regulated in cells is still not clearly understood. At least three types of regulatory mechanisms have been proposed in the literature. First, based on findings that Pfn1 exhibits affinity for membrane phosphoinositides (PPI), and PI(4,5)P_2_ (the most abundant PPI species in cells) micelles can dissociate the Pfn1:actin complex, it has been speculated that phospholipase C-mediated PI(4,5)P_2_ hydrolysis could trigger the release of Pfn1 from the plasma membrane enabling its interaction with actin [7]. Whether this actually occurs in cells has not been examined yet. Second, it was shown that when treated with peroxynitrile, Pfn1 becomes nitrated on a single tyrosine residue at the C-terminus, and this type of modification increases and decreases Pfn1’s affinities for PLP ligands and actin, respectively [8]. It was further demonstrated that activation of inducible Nitric Oxide synthase results in Pfn1 nitration in platelets [9]. Therefore, nitric oxide signaling could potentially modulate ligand interactions of Pfn1. Third, there is also evidence that Pfn1 can be phosphorylated on tyrosine and serine residues. For example, in endothelial cells, VEGFR2 activation leads to Src-mediated phosphorylation of Pfn1 on residue Y129 which increases its affinity for actin [10]. Similarly, activation of the Rho pathway causes ROCK (Rho-associated coiled-coiled kinase)-mediated phosphorylation of Pfn1 on residue S137 [11] impacting its binding to PLP ligands (note that phosphorylation at this site can be also mediated by PKC at least *in vitro* [12]). These studies suggest that acute activation of certain signaling pathways can modulate ligand interactions of Pfn1 through phosphorylation.

The overall goal of this study was to identify other novel phosphorylation events of Pfn1 that can have important functional consequences. We here report that PKA can directly phosphorylate Pfn1 at multiple residues including T89, a residue that is involved in its interaction with actin. Consistent with molecular dynamics simulations, expression studies of phosphomimetic variant of Pfn1 further suggest that T89 is a structurally important residue, phosphorylation of which is likely to influence actin-binding of Pfn1.

## Materials and Methods

### Cell culture

HEK-293 cells (ATCC, CRL-1573) were cultured in DMEM/F12 (1:1) (Life Technologies, Carlsbad, PA, USA) growth medium [10% (v/v) FBS, 100U/mL penicillin, 100μg/mL streptomycin]. HEK-293 cells were maintained on culture dishes (Corning, Corning, NY, USA) coated with type I collagen (BD Biosciences, Franklin Lakes, NJ, USA) for all experiments. MDA-MB-231 cells (ATCC, HTB-26) were cultured in EMEM (Lonza; Basel, Switzerland) growth medium [10% (v/v) FBS, 100U/ml penicillin, 100μg/ml streptomycin]. For activation of cAMP/PKA pathway, cells were serum-starved for 6 hours prior to treatment with 50μM FSK (Sigma, St. Louis, MO) for 5–10 min. In some experiments, cells were pretreated with 10μM H89 (Sigma) or DMSO (vehicle control) for 15 min prior to changing to FSK-containing media in the continued presence of H89 (or DMSO).

### Plasmids and transfection

Phosphomimetic (S57D, T89D, S91D, T92D) and phosphodead (T89A) mutations of *Mus musculus* Pfn1 were introduced into either a bacterial expression vector encoding GST-Pfn1 (a generous gift from Dr. Gerard Marriott, UC Berkeley) or a bicistronic mammalian expression vector (pIRES2-AcGFP1—Clontech, Mountain View, CA, USA) encoding myc-Pfn1 (cloning sites—Xho1, BamH1) using the primers summarized in S1 Table. Plasmid DNA transfections for HEK-293 and MDA-231 cells were performed using XtremeGENE HP transfection reagent (Roche, Basel, Switzerland) and Lipofectamine LTX Plus (Life Technologies, Carlsbad, PA, USA), respectively, according to the manufacturer’s instructions. Gene silencing of Pfn1 was performed as described previously [13]. Pfn1 plasmids were rendered siRNA-resistant by introducing two base-pair silent mutations in the Pfn1-siRNA targeting region.

### *In vitro* Kinase assay

His-tagged Pfn1 (1.5 μg; Cytoskeleton Inc, Denver, CO) was added to kinase buffer [20mM BES (Sigma) pH 7, 20mM EGTA (Fisher Scientific, Waltham, MA), 6mM MgCl2 (Fisher Scientific), 5mM ATP (Sigma), 10mM phosphocreatine (Sigma), 1mM DTT (Roche, Basel, Switzerland)] and incubated with or without 0.5U/μL catalytic subunit of bovine PKA (Sigma) at 30°C for 1 hour with gentle mixing every 15 minutes. The reaction was stopped by boiling the products for 5 minutes in the presence of 2-mercaptoethanol. Reaction products were run on SDS-PAGE and visualized by silver staining.

### In gel trypsin digestion

In gel trypsin digestion was carried out as previously described [14]. PKA treated His-tagged Pfn1 was first subjected to SDS-PAGE. The gels were washed twice with MilliQ water for 5 minutes each and then stained with Coomassie blue [0.1% (w/v) R250 (Fisher Scientific, Waltham, MA, USA), 40% (v/v) ethanol (Decon, King of Prussia, PA, USA), 10% (v/v) Acetic Acid (Fisher Scientific, Waltham, MA, USA)]. After destaining gels with a destaining solution [10% (v/v) ethanol, 7.5% (v/v) acetic acid], His-tagged Pfn1 gel band was excised, washed with HPLC water and destained with 50% acetonitrile (ACN)/25mM ammonium bicarbonate until staining was no longer visible. Gel pieces were dehydrated with 100% ACN, reduced with 10mM dithiothreitol (DTT) at 56°C for 1 hour, followed by alkylation with 55mM iodoacetamide (IAA) at room temperature for 45 min in the dark. Gel pieces were then again dehydrated with 100% ACN to remove excess DTT and IAA, and rehydrated with 20ng/μl trypsin/25mM ammonium bicarbonate and digested overnight at 37°C. The resultant tryptic peptides were extracted with 70% ACN/5% formic acid, vacuum dried and reconstituted in 18μl 0.1% formic acid.

### Tandem mass spectrometry analysis

Proteolytic peptides from in gel trypsin digestion were analyzed by a nanoflow reverse-phased liquid chromatography tandem mass spectrometry (LC-MS/MS). Tryptic peptides were loaded onto a C18 column (PicoChip™ column packed with 10.5cm Reprosil C18 3μm 120Å chromatography media with a 75μm ID column and a 15μm tip, New Objective, Inc., Woburn, MA, USA) using a Dionex HPLC system (Dionex Ultimate 3000, ThermoFisher Scientific, Waltham, MA, USA) operated with a double-split system (Personal communication with Dr. Steve Gygi from Department of Cell Biology, Harvard Medical School) to provide an in-column nano-flow rate (~300 nl/min). Mobile phases used were 0.1% formic acid for A and 0.1% formic acid in acetonitrile for B. Peptides were eluted off the column using a 52 minute gradient (2–40% B in 42 min, 40–95% B in 1 min, 95% B for 1 min, 2% B for 8 min) and injected into a linear ion trap MS (LTQ-XL, ThermoFisher Scientific, Waltham, MA, USA) through electrospray. The LTQ XL was operated in a date-dependent MS/MS mode in which each full MS spectrum [acquired at 30000 automatic gain control (AGC) target, 50ms maximum ion accumulation time, precursor ion selection range of m/z 300 to 1800] was followed by MS/MS scans of the 5 most abundant molecular ions determined from full MS scan (acquired based on the setting of 1000 signal threshold, 10000 AGC target, 100ms maximum accumulation time, 2.0Da isolation width, 30ms activation time and 35% normalized collision energy). Dynamic exclusion was enabled to minimize redundant selection of peptides previously selected for CID. The MS raw files have been deposited to Chorus (https://chorusproject.org/pages/index.html) under the project “Phosphorylation of Pfn1”.

### Peptide identification by database search

MS/MS spectra were searched using the SEQUEST search engine implemented in Proteome Discoverer^™^ software (v. 1.4.0, ThermoFisher Scientific, Waltham, MA, USA) against a UniProt human proteome database (January 2013 release) from the European Bioinformatics Institute (http://www.ebi.ac.uk/integr8). The following modifications were used: static modification of cysteine (carboxyamidomethylation, +57.05 Da), variable modification of methionine (oxidation, +15.99Da) and variable modification of serine/threonine/tyrosine (phosphorylation, +79.97Da). The mass tolerance was set at 1.4Da for the precursor ions and 0.5Da for the fragment ions. Peptide identifications were filtered using PeptideProphet^™^ and ProteinProphet^®^ algorithms with a protein threshold cutoff of 99% and peptide threshold cutoff of 95% implemented in Scaffold^™^ (Proteome Software, Portland, Oregon, USA).

### Two-dimensional gel electrophoresis (2D-GE)

Cells were scraped in the presence of 2D lysis buffer [2M urea (Fisher Scientific), 7M thiourea (Life Technologies, Carlsbad, PA, USA), 4% (w/v) CHAPS (Sigma), 50mM DTT] and collected into a bead-beater tube (Biospec, Bartlesville, OK, USA) containing 50mg Glass Beads (Sigma). Isoelectric focusing was performed using the Zoom IPG Runner System (Life Technologies) according to manufacturer’s instructions with modification. Briefly, lysates were reconstituted in rehydration buffer [final concentrations: 2M urea, 7M thiourea, 4% (w/v) CHAPS, 50mM DTT, 0.5% (v/v) carrier ampholytes (Life Technologies), 0.005% (w/v) bromophenol blue (Fisher Scientific), 50–150μg protein]. IPG strips underwent isoelectric focusing using the following program: 175V for 45 minutes; linear ramp 175-2000V over 45 minutes; 2000V for 45 minutes. IPG strips were briefly washed with running buffer [25mM Tris (pH 8.3), 192mM glycine, 0.1% (w/v) SDS] and sealed on Tris-HCl polyacrylamide gels with running buffer containing 0.5% agarose (Life Technologies) and 0.005% (w/v) bromophenol blue before performing electrophoresis in the second dimension followed by immunoblotting.

### Immunoblotting

Total lysates were prepared by extracting cells with modified RIPA buffer (25mM Tris—HCl (pH 7.5), 150mM NaCl, 1% (v/v) NP-40, 5% (v/v) glycerol, 1mM EDTA, 50mM NaF, 1mM sodium pervanadate, and protease inhibitors). In some experiments, SDS and/or urea were added to RIPA buffer at final concentrations of 2% and 6M, respectively. Immunoblotting conditions for the various antibodies were: monoclonal GFP (Clontech, Mountain View, CA, USA, 1:3000), monoclonal Pfn1 (Abcam, Cambridge, England, 1:3000), monoclonal actin (BD Biosciences, Frankin Lakes, NJ, USA. 1:1000), monoclonal myc (Sigma, 1:3000), monoclonal α-tubulin (Sigma, 1:3000), monoclonal VASP (BD Biosciences, Frankin Lakes, NJ, USA, 1:1000) and monoclonal p27^kip1^ (BD Biosciences, Franklin Lakes, NJ, USA, 1:2000).

*Phalloidin staining*—Cells were washed with DPBS, fixed with 3.7% formaldehyde for 15 min, permeabilized with 0.5% Triton X-100 for 5 min and then blocked with 10% goat-serum for 1 h at room temperature. Cells were incubated with rhodamine-phalloidin (Life Technologies) for an hour at room temperature. Stained cells were washed two times with PBS containing 0.02% tween, two times with PBS, and then once with distilled water before mounting on slides for imaging using a 60X oil-immersion objective on an Olympus IX71 inverted microscope.

### GST-pull down assay

Bacteria carrying various forms of GST-Pfn1 were grown at 37°C under constant agitation in the presence of antibiotics until the optical density at 600 nm was between 0.6 and 0.9. Cells were induced with 0.1mM isopropyl-β-D- thiogalactopyranoside (IPTG) for 3 hours, and extracted with lysis buffer (25mM Tris-base (pH 7.4), 150mM NaCl, 1% NP-40, 1mM EDTA, 5% glycerol) followed by sonication. Bacterial extracts were clarified at 18000g for 30 minutes before incubating with glutathione-agarose beads (Sigma) for 2 hours at 4°C with constant rotation. Beads were washed 5 times with lysis buffer and subjected to pull-down assay with HEK-293 lysate overnight at 4°C. After washing the beads 5 times with lysis buffer, protein was eluted by boiling beads for 5 minutes in the presence of 2-mercaptoethanol and subjected to SDS-PAGE and immunoblot analyses.

### Molecular Dynamics Simulation

A homology model of *Mus musculus* Pfn1 was created from a crystal structure of bovine Pfn1 bound to actin (PDB 2BTF). The model was created by manually performing the following mutations using Pymol: N9S, N41S, I49V, I100V, and M122L. All mutations could be performed without introducing significant steric clashes. Additional Pfn1 models were created by mutating T89 to either alanine or aspartic acid to mimic phospho-dead and phosphorylated states as well as to phosphothreonine. All simulations were performed with GPU accelerated Amber version 14 with the ff14sb force field [15]. Parameters for phosphothreonine were taken from a previously published study [16]. The homology modules were solvated to form an octahedral TIP3P water box that extends 12 Å beyond the protein. The system was neutralized with Na+ or Cl- ions as needed. The system then underwent two rounds of minimization. A 100ps constant volume run was performed with weak positional restraints on the protein during which the temperature was warmed from 0°K to 300°K followed by an unrestrained constant temperature and pressure equilibration run of 100pS. Simulations were performed using the particle mesh Ewald (PME) method with a non-bonded cutoff of 10Å. Constant pressure periodic boundaries and isotropic position scaling were used to maintain a pressure of 1 atm. Langevin dynamics were used for temperature control. Three separate simulations of each model were run with different random seeds and every simulation was run for 300ns, resulting in 900ns of simulation per a model. Analysis of the simulations was performed using MDAnalysis [17].

### Statistics

We performed either Student’s t-test or one-way ANOVA (when more than two groups were involved) for statistical comparison of the means between the different groups and p<0.05 was considered to be statistically significant.

## Results

### PKA phosphorylates Pfn1 on multiple residues *in vitro*

**Fig 1:**
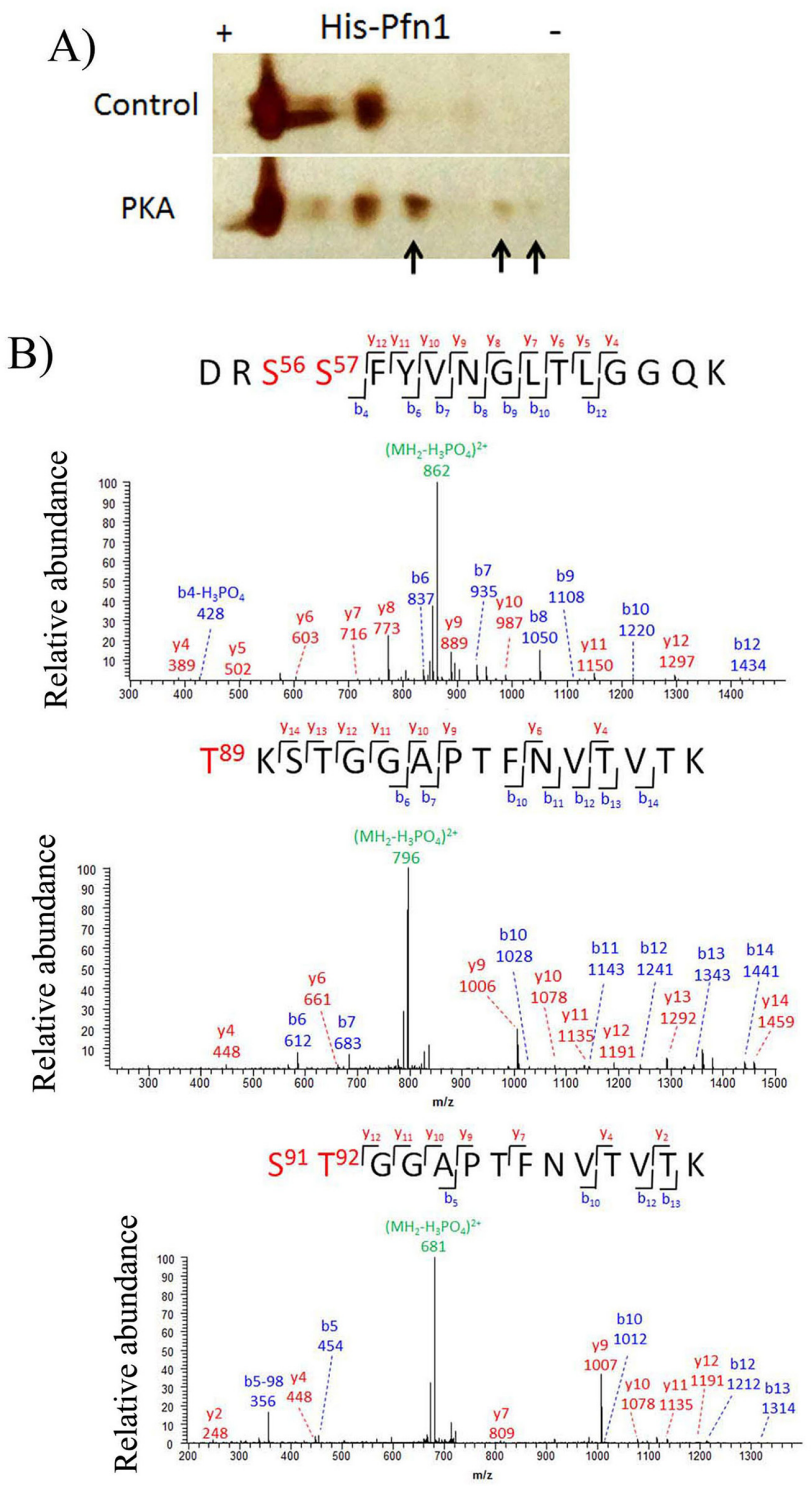
Pfn1 can be phosphorylated by PKA on multiple residues. **A)** His-tagged Pfn1 was treated with buffer containing ATP with or without PKA followed by 2D electrophoresis and visualized by silver staining (isoelectric focusing was conducted using a pH 4–7 IPG strip). Phosphorylation spots are marked by arrows. **B)** Mass-spectrometry based detection of phosphopeptides from PKA-treated His-Pfn1 reveled Pfn1 phosphorylation on three residues (S56/S57, T89, and S91/T92).

To predict potential phosphorylation sites of Pfn1and the likely kinases associated with these sites, we performed sequence analyses of both human and mouse Pfn1 using two different bioinformatics tools (NetphosK, KinasePhos), the results of which are summarized in S2 Table. As per these analyses, the abundance of predicted phosphorylation sites is higher for serine/threonine residues than tyrosine residues, and among the various candidate serine-threonine kinases considered, PKA and PKC, the two AGC-family kinases, are associated with the most number of predicted phosphorylation sites. PKC-mediated phosphorylation of Pfn1 on S137 residue has been characterized previously [11,12]. Based on alterations in post-translational modification patterns of Pfn isoforms in astrocytes upon treatment with FSK (an activator of cyclic AMP-PKA pathway), it has been recently suggested that PKA may be involved in post-translational regulation of Pfn isoforms [18]. Whether Pfn1 is a phosphorylation substrate of PKA however remains unknown. Therefore, as a potential strategy to uncover novel phosphorylation sites of Pfn1, we performed *in vitro* phosphorylation assays using recombinant His-tagged Pfn1 and the catalytic subunit of PKA. We observed acidic shift of a significant population of His-Pfn1 (unmodified isoelectric point ~6.5) as evident from several new charged states demonstrating that PKA is capable of directly phosphorylating Pfn1 at multiple residues at least in vitro (Fig 1A). To identify these phosphorylation sites of Pfn1, PKA-treated His-Pfn1 was trypsin-digested and subjected to LC-MS/MS analysis. A number of Pfn1 peptides covering 53% of Pfn1 sequences were identified, including three singly phosphorylated peptides. Fragmentation patterns of these phosphopeptides were able to narrow down the potential phosphorylation sites to 3 serine/threonine residues: S56/S57, T89, S91/T92 (Fig 1B). Note that the MS/MS spectra are not able to locate the exact modification sites for some phosphopeptides due to the presence of multiple serine/threonine residues (e.g. S56/S57 and S91/T92) and the absence of signature fragment ions for site specificity. Multiple sequence alignment of Pfn1 in vertebrates revealed a high degree of conservation of S56, S57, T89, S91, T92 and the surrounding residues (*data not shown*). As the protein coverage was incomplete, we could not rule out the possibility of other PKA-phosphorylation sites of Pfn1.

### Activation of cAMP/PKA pathway induces acidic charge shift of Pfn1 in non-neuronal cells

**Fig 2:**
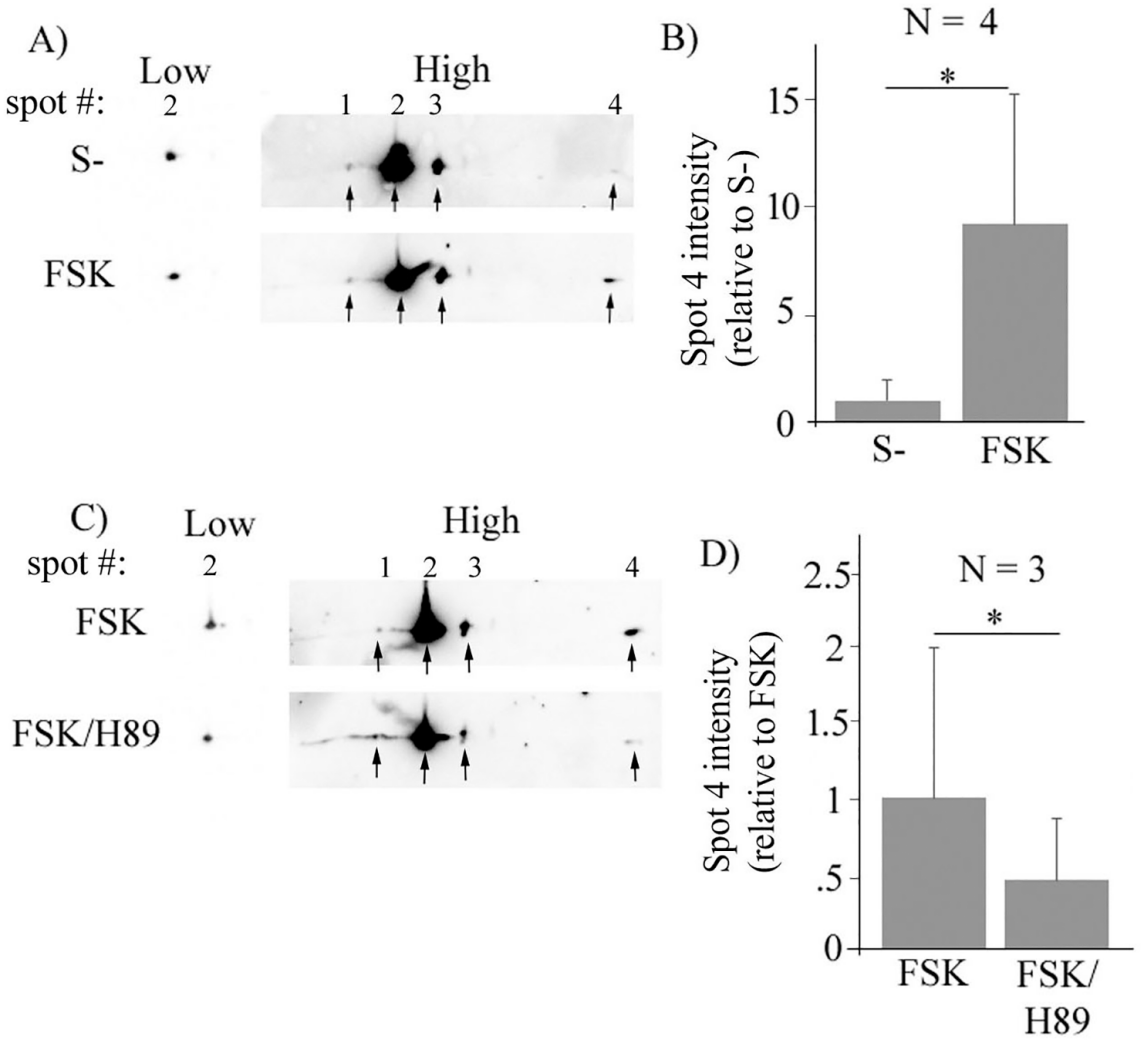
Pfn1 can be post-translationally modified in a PKA-dependent manner in HEK-293 cells. **A)** Lysates prepared from myc-Pfn1-expressing HEK-293 cells were resolved on 4–7 pH gradient IEF gels and then immunoblotted with anti-myc antibody to reveal the isoelectric profile of myc-Pfn1 in serum-starved (control: S-) vs FSK-stimulated conditions—two different exposure blots are shown. **B)** A bar graph summarizing the relative average intensity of the spot representing the most negatively charged form of myc-Pfn1 (spot #4) in control vs FSK-stimulated conditions. **C-D)** Isoelectric profiles of myc-Pfn1 in FSK-stimulated cells with or without H89 treatment (*panel C*), and the corresponding quantifications of the intensity of spot #4 (*panel D*). N denotes the number of independent experiments (*: p<0.05).

Next, to determine whether activation of cAMP-PKA pathway has any effect on the isoelectric profile of Pfn1 in non-neuronal cells, HEK-293 cells overexpressing myc-Pfn1 were either serum-starved or acutely stimulated by FSK before performing 2D-GE analyses of cell extracts. We chose to investigate FSK’s effect on isoelectric profile of myc-Pfn1 instead of endogenous Pfn1 for two main reasons. First, we reasoned that small changes in the isoelectric profile would be easier to detect in an overexpressed condition. Second, our pilot experiments demonstrated that myc-Pfn1 (theoretical PI: 6.3) is much better resolvable than endogenous Pfn1 (theoretical PI—8.3) on IEF, likely owing to its acidic nature (it circumvents the problem of reducing agent DTT migrating away the protein). We found that even under serum-starved condition, myc-Pfn1 exists in multiple charged states as evident from three distinct spots (spots #1 through 3) with spot #2 representing the predominant pool of myc-Pfn1 and an extremely weak fourth spot (#4) representing the most negatively charged form of the protein (Fig 2A). The most obvious FSK-dependent change in the 2D profile of myc-Pfn1 was a prominent increase in the intensity of spot #4 (Fig 2A) reflecting an acidic charge shift involving a small pool of Pfn1, a finding that is consistent with PKA’s ability to phosphorylate Pfn1 *in vitro*. As spot #2 accounted for >95% of total pool of myc-Pfn1, we used the relative intensities of spot #2 at a very low exposure (that does not saturate the signal of this spot) between control and FSK-treatment groups as a correction factor for loading control in these experiments to quantify the fold-changes in the intensity of spot #4 in response to FSK. Using this correction factor, we estimated that FSK treatment led to approximately 9-fold (data summarized from 4 independent experiments) increase in the intensity of spot #4 (Fig 2B). In a set of follow-up experiments, we examined the isoelectric profile of myc-Pfn1 in FSK-stimulated HEK-293 cells with or without pharmacological inhibition of PKA by H89. As expected, H89 pretreatment significantly reduced the intensity of spot #4 (Fig 2C and 2D) further confirming that the most negatively charged pool of Pfn1 in FSK-treated condition is PKA-dependent. Overall, these cell-based experimental results are in agreement with PKA’s effect on Pfn1 *in vitro*.

### T89 phosphorylation affects the biochemical characteristics of Pfn1

**Fig 3:**
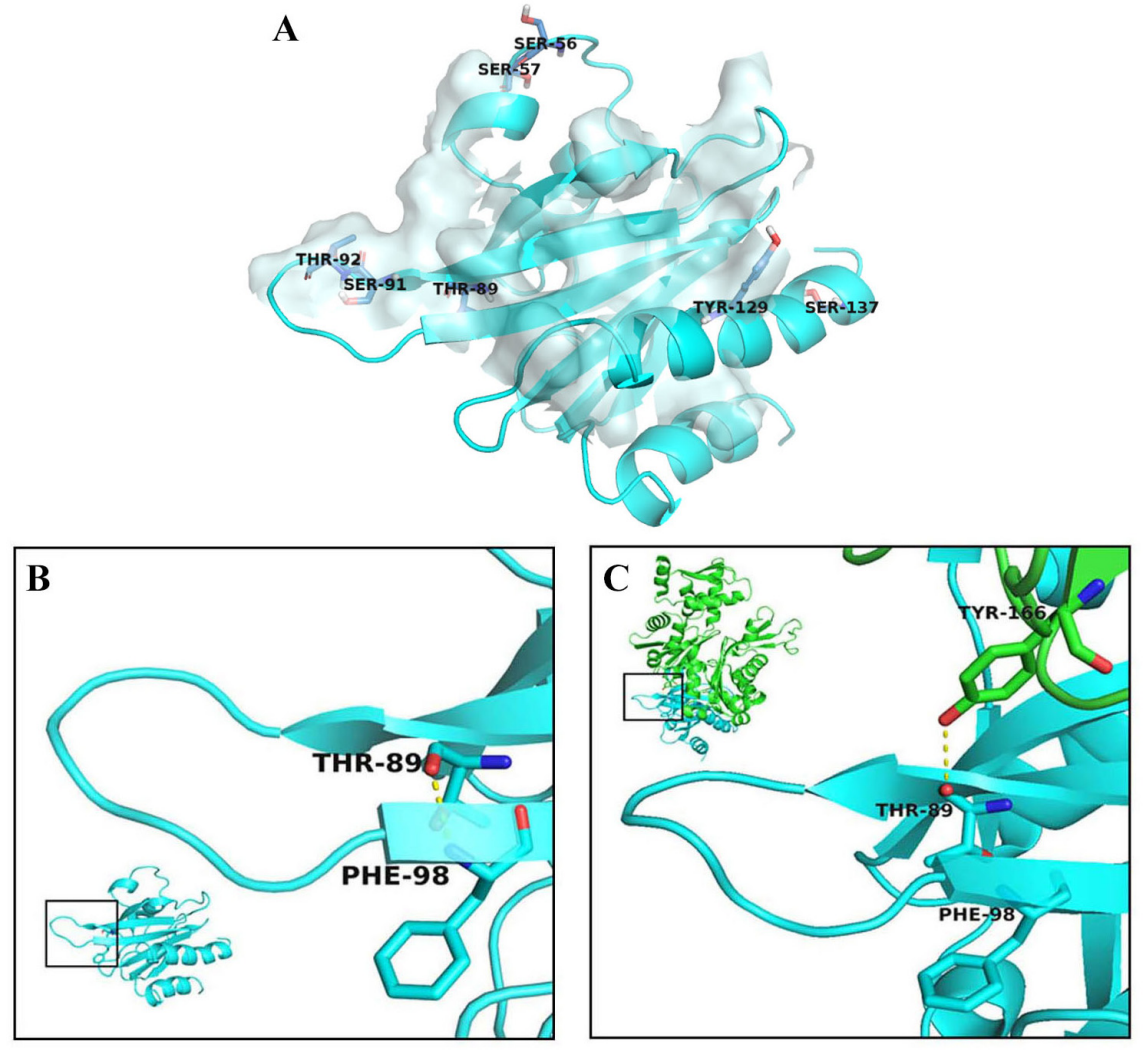
Molecular interactions of T89 of Pfn1. **A)** Actin-interacting surface of Pfn1 (Pfn1: blue; actin: grey); phosphorylatable residues of Pfn1 are indicated as sticks. **B)** In unbound Pfn1 (PDB 1PFL) T89 forms an intramolecular hydrogen bond with F98. **C)** In the Pfn1-actin complex (PDB 2BTF) an intermolecular hydrogen bond (dashed yellow line) is observed between T89 of Pfn1 (blue) and Y166 of actin (green).

**Fig 4:**
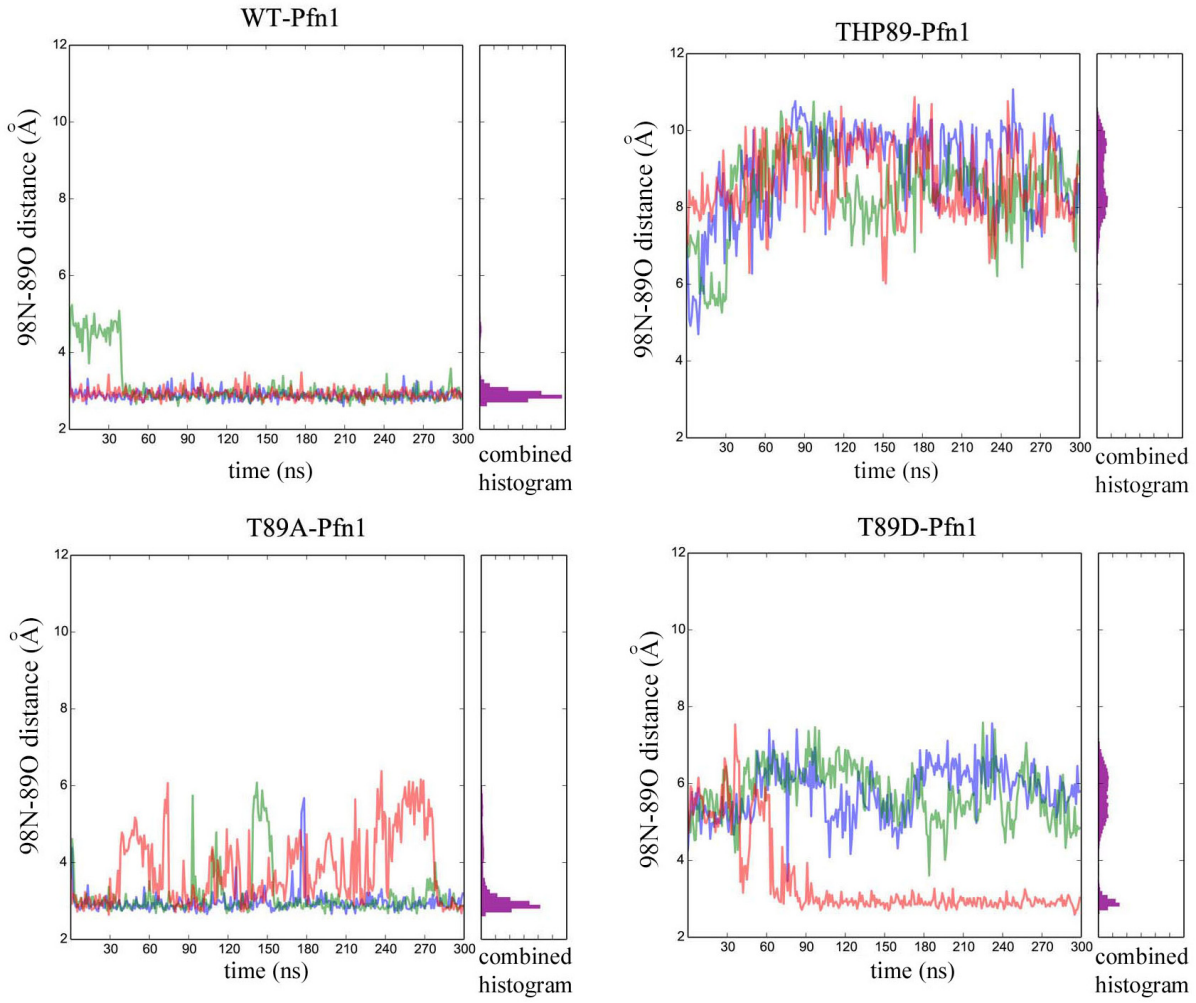
Molecular Dynamics Simulation predicting the effect of T89 phosphorylation on the stability of intramolecular 98N-89O bond. The stability of this intramolecular 98N-89O bond over the course of three 300ns molecular dynamics simulations of monomeric Pfn1 are shown for homology models based on the bound structure for WT, T89A, T89D and phosphorylated T89 (THP89) variants of Pfn1 (each color represents an individual simulation). The histograms alongside the line graphs summarize the results of all three simulations for each variant of Pfn1.

Phosphopeptides of Pfn1 involving S56, S57, T89, S91, T92 have been previously found in global proteomic screens of mitotic cells, cancer and immortalized T cell lines, and endothelial cells suggesting that Pfn1 can be phosphorylated on these residues at least in some cell types under certain conditions [10,19,20]. However, the functional significances of these post-translational modifications of Pfn1 are not known. We were particularly interested in the functional consequence of T89 phosphorylation since in a bound conformation with actin (PDB 2BTF), the backbone oxygen of T89 of Pfn1 makes an intermolecular hydrogen bond with Y166 of actin (Fig 3A and 3B) making T89 as a potentially interesting site for further exploration of possible regulation of the Pfn1:actin interaction. In the monomeric, unbound (i.e when not interacting with any other ligand) structure of Pfn1 (PDB 1PFL), the backbone oxygen of T89 forms an intramolecular hydrogen bond interaction (distance ≤ 3.0 Å) with the backbone nitrogen of F98 (Fig 3C). The T89:F98 hydrogen bond marks the transition between the loop defined by residues 89–98 and a beta sheet. Due to the geometry of the interactions, the intramolecular T89 (Pfn1):F98 (Pfn1) and intermolecular T89 (Pfn1):Y166 (actin) hydrogen bonds are mutually exclusive. Given the importance of the intramolecular T89:F98 interaction of Pfn1 in determining its interaction with actin, we asked whether phosphorylation of T89 could potentially affect the 98N-89O hydrogen bond. To address this question, we performed molecular dynamics simulations (MDS) on monomeric homology models of wild-type (WT)-Pfn1 with or without phosphorylated-T89, T89D-Pfn1 (T89 was replaced by an aspartic acid, a strategy that is commonly used to artificially mimic a phosphorylated state of an amino acid) or T89A (T89 was replaced by an alanine to simulate a corresponding non-phosphorylatable version). All simulations were started with a conformation where T89:F98 interaction was not initially formed. We assessed the stability of the intramolecular 98N-89O hydrogen bond over the course of three independent 300-ns simulations. The time-dependent fluctuation of 98N-89O distance for each of the three simulations and a histogram summarizing the results of all three simulations for the four variants (WT, WT-phosphoT89, T89A and T89D) of Pfn1 are depicted in Fig 4. In all three simulations, the WT protein quickly established a stable intramolecular T89:F98 (distance ≤ 3.0 Å) bond. By contrast, the intramolecular hydrogen bond was not formed in any of the three THP89 (phosphorylated T89) simulations, suggesting that phosphorylation of T89 causes an extreme destabilization of the intramolecular T89:F98 interaction. Even though the histogram pattern of the T89D simulation was not exactly identical to that of THP89, in two out of three simulations T89D-Pfn1 also completely failed to establish the hydrogen bond throughout the course of the simulation suggesting that T89D-Pfn1 is a reasonable mimic for T89-phosphorylated Pfn1. Similarly, the histogram pattern of the T89A mutant, although not identical, was also fairly similar to that of the WT protein suggesting that this non-phosphorylatable mutant also stabilizes the interaction. The differences in histogram patterns between T89A and WT or THP89 and T89D simulations were not totally unexpected since aspartic acid and alanine are not structurally identical to phosphorylated and unphosphorylated threonine, respectively. In summary, these simulation results predicted that the intramolecular T89:F98 interaction of Pfn1 could be sensitive to the phosphorylation status of T89. Since T89 (Pfn1):F98 (Pfn1) and T89 (Pfn1):Y166 (actin) interactions are mutually exclusive, one possible consequence of destabilization of the T89:F98 interaction upon T89 phosphorylation is enhanced stability of the T89 (Pfn1):Y166 (actin) interaction when Pfn1 is in a complex with actin and, therefore, a potential stronger affinity of Pfn1 for actin.

**Fig 5:**
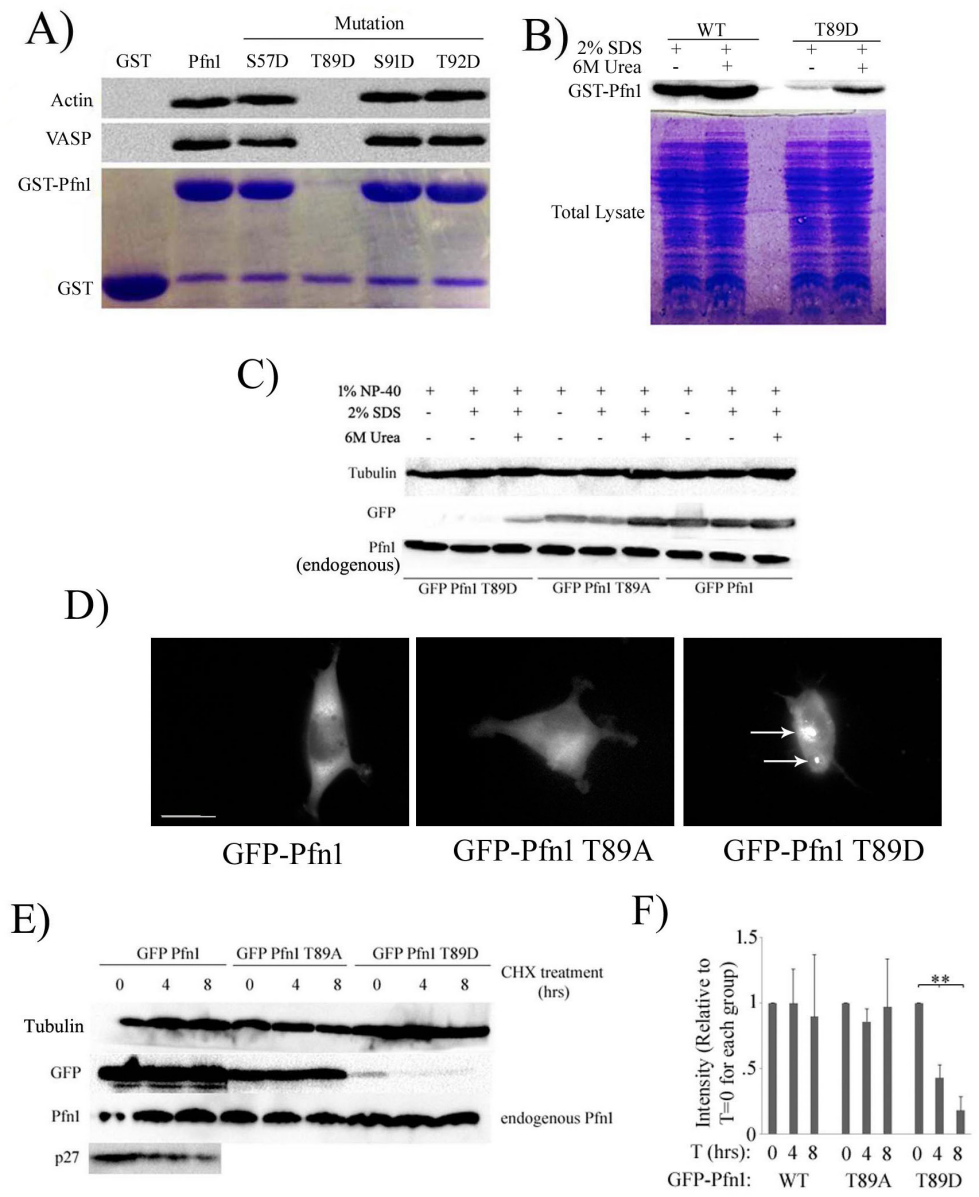
Effect of site-specific phosphorylation on biochemical characteristics of Pfn1. **A)** Pull-down assays of indicated GST-tagged Pfn1 constructs with HEK-293 cell lysate were run on an SDS-PAGE and immunoblotted with anti-actin and anti-VASP antibodies (GST was used as a negative control). Coomassie stain in parallel confirms comparable amounts of GST-tagged proteins in the pull-down assay. Note that virtually negligible amount of T89D-Pfn1 was found to be immobilized on glutathione-linked agarose beads. **B)** Bacteria expressing indicated GST-tagged Pfn1 constructs were lysed with either non-denaturing (containing 1% NP-40) or denaturing (containing 1% NP-40, 2% SDS for one buffer and the other with 6M urea in addition) extraction buffers. Bacterial lysates were immunoblotted with anti-Pfn1 antibody to demonstrate that T89D-Pfn1 is insoluble in non-denaturing lysis buffer. **C)** HEK-293 cells expressing indicated EGFP-fused Pfn1 constructs were lysed with either non-denaturing (containing 1% NP-40) or denaturing (containing 1% NP-40, 2% SDS for one buffer and the other with 6M urea in addition) extraction buffers. HEK-293 lysates were immunoblotted with anti-GFP antibody to demonstrate that GFP-T89D-Pfn1 is also insoluble in non-denaturing lysis buffer. Note that endogenous Pfn1 level is not affected by expression of any of the ectopic Pfn1 constructs and extractable completely in non-denaturing lysis buffer. **D)** Fluorescence images of HEK-293 cells expressing indicated EGFP-fused Pfn1 constructs show that EGFP-Pfn1-T89D causes clustering of this fusion protein as indicated by the arrows. Scale bar represents 20 μm. **E)** HEK-293 cells expressing indicated EGFP-fused Pfn1 constructs were treated with CHX for up to 8 hours. Cell lysates prepared at different time-points after CHX addition were immunoblotted with the indicated antibodies. T89D-Pfn1 undergoes rapid protein degradation while WT- and T89A-Pfn1 are stable over that period of time, similar to the characteristic of endogenous Pfn1 (degradation of p27kip1, a cell-cycle protein that undergoes rapid turnover, validates CHX efficacy). Tubulin blot serves as the loading control. **F)** The bar graph summarizes quantification of the time-dependent changes in the expression of the indicated GFP-Pfn1 constructs following CHX treatment in HEK-293. Data was summarized from 3 independent experiments (** indicates p < .01).

We next performed GST-pull-down assays of bacterially expressed forms of either WT (control)- or various phosphomimetic Pfn1 using HEK-293 cell lysate to qualitatively assess the changes in Pfn1:actin binding conferred by T89D substitution. To our surprise, the pull-down assay failed for T89D-Pfn1 as this mutant protein was largely insoluble in non-denaturing lysis buffer and therefore could not be extracted from the bacteria (Fig 5A). T89D-Pfn1 was extractable from bacteria only under denaturing lysis buffer and in the presence of urea (Fig 5B), a condition that is unfortunately non-permissive for binding studies. The protein insolubility issue was specific to T89D substitution since phosphomimetic Pfn1 constructs involving all other PKA sites (S57, S91 and T92) were extractable in non-denaturing lysis buffer and showed ability to bind actin and VASP (a PLP ligand of Pfn1) similar to the extent of WT Pfn1, at least, on a qualitative scale (Fig 5A). Even in mammalian HEK-293 cells, T89D-Pfn1, when transiently expressed as an EGFP-tagged protein, was barely extractable in non-denaturing lysis buffer and could only be extracted efficiently when both SDS and urea were present in the lysis buffer; WT- and T89A-Pfn1 were fully extractable in mild non-denaturing lysis buffer as expected (Fig 5C). Similarly, T89D-Pfn1 was hardly extractable in non-denaturing lysis buffer when expressed as a myc-tagged protein (S1 Fig). Therefore, protein insolubility of T89D-Pfn1 does not appear to be either epitope-tag- or cell-type (bacterial vs mammalian)-specific effect. Fluorescence microscopy of HEK-293 cells transfected with the various EGFP-Pfn1 constructs revealed that nearly all cells expressing EGFP-T89D-Pfn1 formed prominent protein aggregates of EGFP-Pfn1 whereas neither WT nor T89A overexpressors demonstrate EGFP-Pfn1 clustering, a result that is consistent with the insolubility characteristics of T89D-Pfn1 (Fig 5D). At the protein level, expression of EGFP-Pfn1-T89D was always found to be much lower than either of the other two exogenous (WT or T89A) forms of Pfn1 (Fig 5C). This was not due to differences in the levels of transfected DNA since mRNA levels of all three EGFP-fused Pfn1 constructs were comparable (S2 Fig). Note that endogenous Pfn1 expression was not affected by overexpression of any of these ectopic Pfn1 constructs (Fig 5C). When we blocked new protein synthesis in HEK-293 cells by cycloheximide (CHX) treatment, both WT- and T89A-variants of EGFP-Pfn1 showed no detectable change in the protein level over a period of 8 hours but the expression level of T89D-Pfn1 declined substantially within 4 hours of CHX treatment (Fig 5E and 5F). These results demonstrate that T89D substitution accelerates the protein turnover of Pfn1. Folding of proteins into alternative three-dimensional conformation can lead to protein aggregation, insolubility and marking for proteolytic degradation. As T89D-Pfn1 exhibits all of these hallmarks, it indirectly suggests that T89D substitution causes a significant conformational change in Pfn1, an interpretation that is consistent with our *in silico* prediction of extreme destabilization of the intramolecular T89:F98 interaction upon T89 phosphorylation. Therefore, T89 appears to be a structurally important residue of Pfn1 that can be modified by phosphorylation.

Since a direct biochemical assessment of the effect of T89 phosphorylation on actin-binding of Pfn1 was not possible due to protein insolubility issues, we considered two indirect approaches. First, we performed the standard pyrene-actin polymerization assay to determine whether PKA-mediated phosphorylation has any effect on Pfn1’s ability to inhibit actin polymerization. In the absence of PKA, Pfn1 inhibited actin polymerization by ~30% when added to actin at a 1:1 molar ratio. Unfortunately, pre-incubation with PKA (mimicking the *in vitro* kinase assay condition) made little to no difference in the overall effect of Pfn1 on the kinetics of actin polymerization (data not shown). This was not totally unexpected because if only a very small fraction of Pfn1 is modified on T89 by PKA, the resulting effect would be hard to detect in a bulk polymerization assay such as the pyrene-actin assay. It is possible that PKA-mediated phosphorylation on other residues may have a confounding effect masking the effect of T89 phosphorylation.

**Fig 6:**
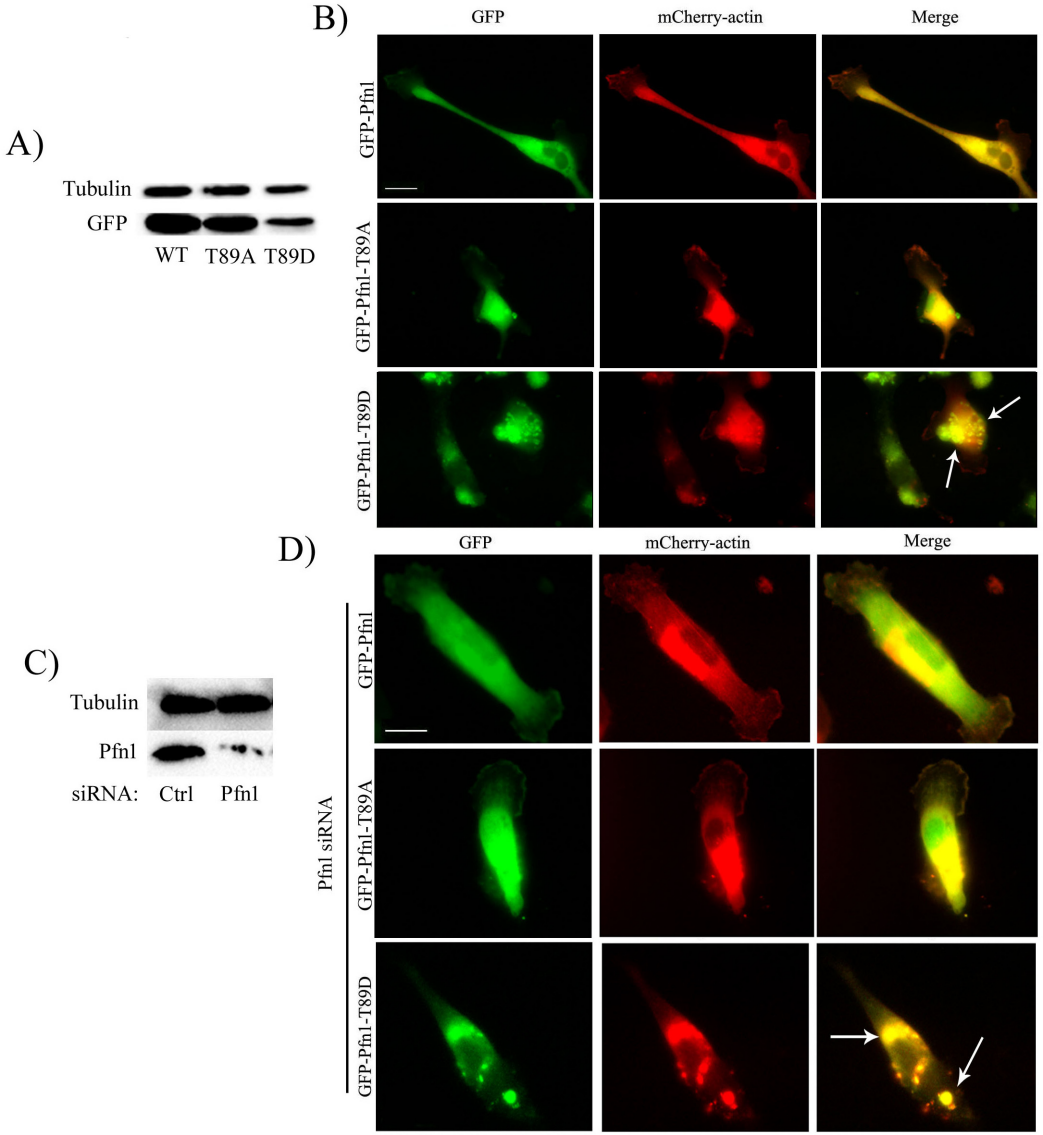
T89D-Pfn1 co-clusters with actin in cells. **A)** Lysates prepared from MDA-231 cells expressing indicated EGFP-fused Pfn1 constructs were immunoblotted with anti-GFP antibody to show the relative levels of various EGFP-Pfn1 constructs (tubulin blot serves as the loading control). **B)** Fluorescence images of MDA-231 cells expressing indicated EGFP-fused Pfn1 and mCherry-actin demonstrate mCherry-actin clustering at the sites of T89D-Pfn1 aggregates (arrows; Scale bar—20 μm). **C)** Fluorescence images of MDA-231 cells co-expressing mCherry-actin and EGFP-Pfn1-T89D, and treated with Pfn1 siRNA reveal mCherry-actin:T89D-Pfn1 co-clusters (arrows; Scale bar—20 μm). Pfn1 immunoblot alongside confirms near complete loss of endogenous Pfn1 expression in Pfn1-siRNA treated cells when compared against control siRNA transfected cells (tubulin blot—loading control).

**Fig 7:**
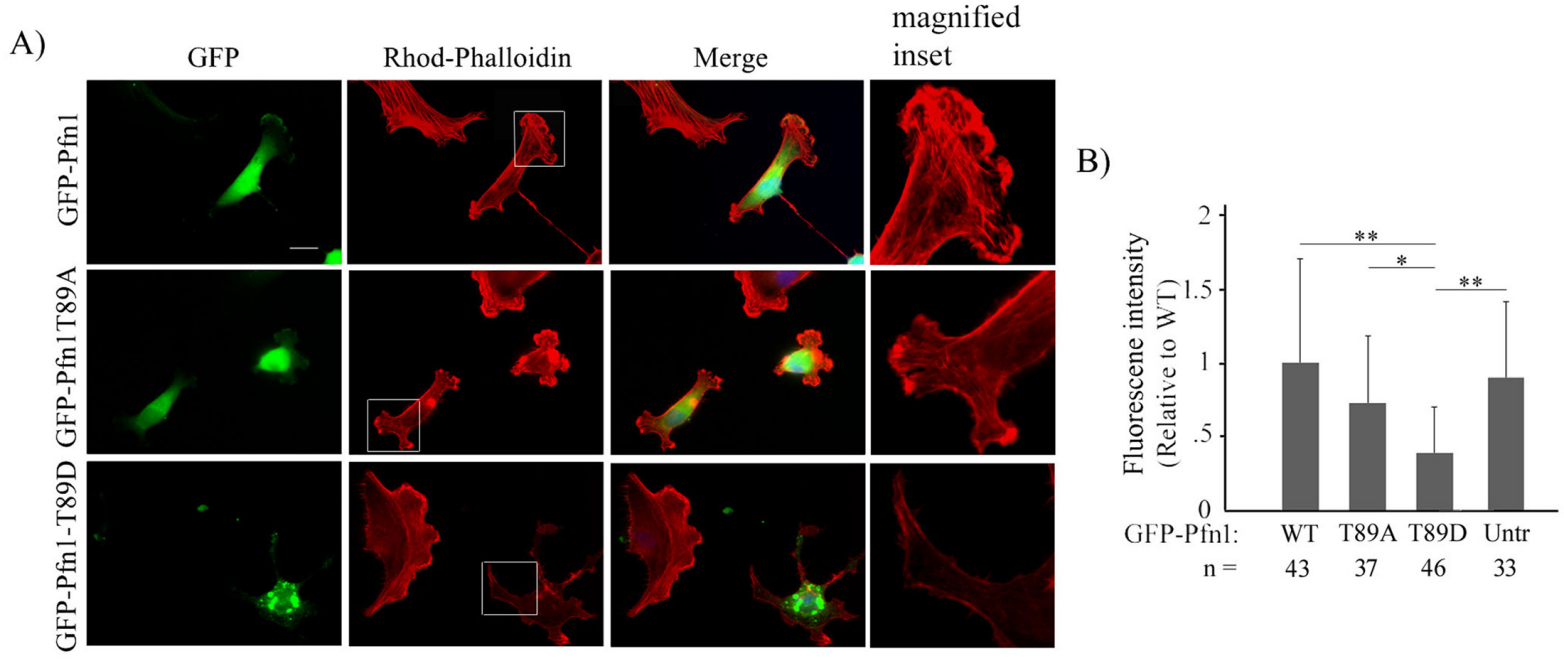
Overexpression of T89D-Pfn1 has a robust effect on actin cytoskeleton in MDA-231 cells. **A)** Fluorescence images of MDA-231 cells expressing indicated EGFP-fused Pfn1 constructs and stained with rhodamine-phallodin staining. Scale bar represents 20 μm. **B)** A bar graph summarizing the average rhodamine-phalloidin fluorescence intensity/cell for T89A-Pfn1 and T89D-Pfn1 expressors relative to that of WT-Pfn1 expressing and untransfected cells. ‘N’ indicates number of cells analyzed for each group pooled from 3 independent experiments (** indicates p < .01; * indicates p < .05).

Therefore, as an alternative strategy, we investigated the effect of constitutive T89 phosphorylation of Pfn1 on actin polymerization in cells. First, we transiently overexpressed mCherry-actin along with either WT- or T89A- or T89D- variants of Pfn1 (all constructs were EGFP-fused) in MDA-MB-231 (MDA-231) breast cancer cells. As in HEK-293 cells, T89D-Pfn1 a) expressed at a much lower level than either WT- or T89A-Pfn1 (Fig 6A), and b) formed prominent aggregates (Fig 6B) in MDA-231 cells. Interestingly, mCherry-actin was also found to be strikingly concentrated at T89D-Pfn1 clusters (Fig 6B). Clustering of mCherry-actin was not observed in cells overexpressing either WT or T89A-Pfn1. We considered two scenarios by which T89D-Pfn1 overexpression could induce actin clusters. One possibility is that T89D-Pfn1 directly binds to actin and co-clusters actin at the sites of their aggregation. As there is also experimental evidence of Pfn1’s ability to oligomerize at least *in vitro* [21], an alternative possibility is that T89D-Pfn1 somehow oligomerizes with endogenous Pfn1 and in turn recruits actin bound to endogenous Pfn1. To determine whether T89D-Pfn1:actin co-clustering requires the action of endogenous Pfn1, we re-assessed T89D-Pfn1:actin co-clustering in MDA-231 cells when endogenous Pfn1 expression was selectively suppressed by siRNA transfection. Even in a near complete absence of endogenous Pfn1 expression, prominent co-clustering of mCherry actin and T89D-Pfn1 was observed (Fig 6C). These data suggest that in an overexpression setting (i.e in the presence of endogenous Pfn1), co-clustering of mCherry-actin and T89D-Pfn1 is most likely mediated by a direct interaction between T89D-Pfn1 and actin rather than involving an indirect action of endogenous Pfn1. In parallel experiments, we also found that overexpression of only T89D-Pfn1 but not WT- or T89A-Pfn1 has a significant impact on the average level of polymerized actin (as measured by rhodamine-phalloidin fluorescence on a cell-by-cell basis) in MDA-231 cells (Fig 7A and 7B; note that the overall expression of actin is not affected by T89D-Pfn1 overexpression (S3 Fig)). T89D-Pfn1 overexpression resulted in a robust 60% decrease in average F-actin content/cell when compared to the GFP control; there was no significant difference in average F-actin level between either of WT- or T89A-Pfn1 overexpressor. Inhibitory action of T89D-Pfn1 on actin polymerization in cells, taken together with the actin clustering ability of T89D-Pfn1 even in the absence of endogenous Pfn1, is consistent with intact actin-binding of T89D-Pfn1 (or T89 phosphorylated Pfn1) as predicted by MDS. Despite the low expression level of T89D-Pfn1 compared to either WT—or T89A-Pfn1, a reduction in actin polymerization specifically in the setting of T89D-Pfn1 overexpression may suggest that T89D-Pfn1 has a dominant negative effect. This is clearly possible if T89D-Pfn1 has a stronger affinity for the actin monomer than endogenous Pfn1 (as predicted by MDS) and is thereby able to competitively inhibit the endogenous Pfn1:actin interaction.

### T89 may be an *in vivo* modification site of Pfn1 in the basal state

**Fig 8:**
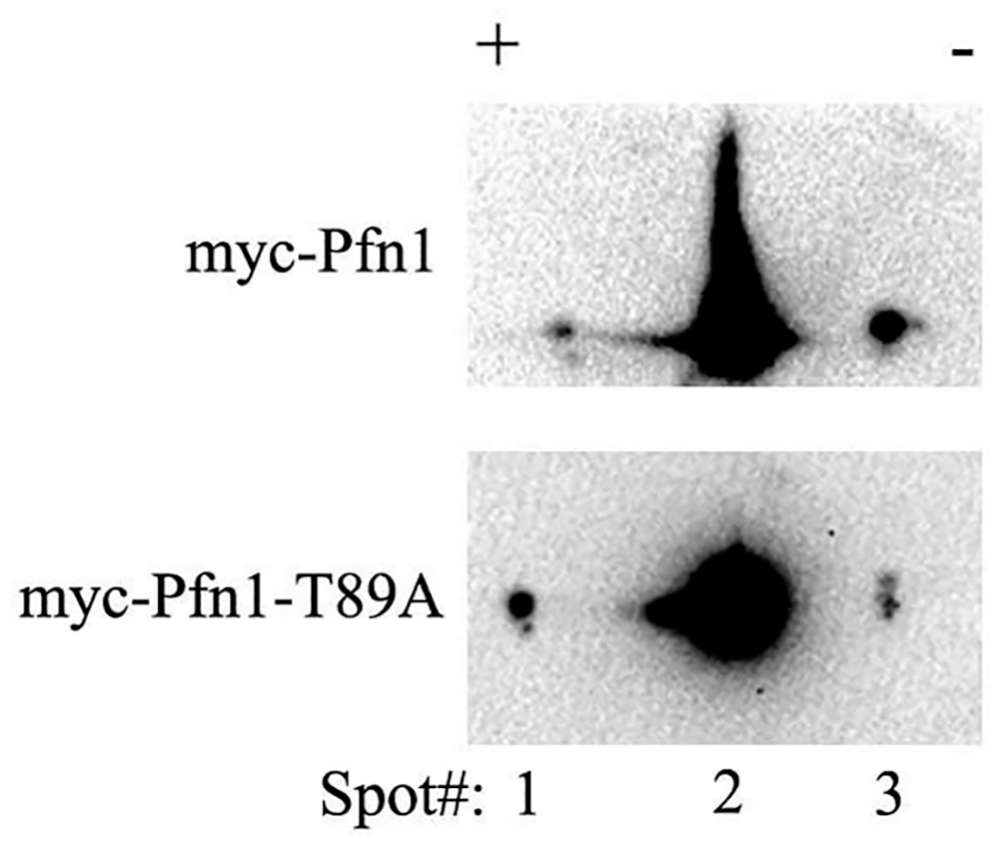
Effect of non-phosphorylatable amino-acid substitution at T89 on post-translational modification profile of Pfn1 in cells. 2D-GE of lysates from HEK-293 cells expressing either myc-Pfn1 or myc-Pfn1-T89A demonstrate that alanine substitution at T89 results in a basic shift of a small portion of myc-Pfn1.

We finally asked whether any subcellular pool of Pfn1 is post-translationally modified on T89 even under the basal condition. Unfortunately, we were unable to directly confirm T89 phosphorylation of Pfn1 by mass-spectrometry of immunoprecipitated myc-Pfn1 from HEK-293 cells. We speculated that this could be either due to a) a possibility of only a very negligible fraction of Pfn1 that is being modified at the T89 residue, and/or b) detergent insolubility of T89-phosphorylated Pfn1 (as suggested by the biochemical characteristics of phosphomimetic T89D-Pfn1) thus precluding T89-phosphorylated Pfn1 from the immunoprecipitated sample. Therefore, we adopted an indirect approach where we expressed either WT- or non-phosphorylatable T89A forms of Pfn1 as myc-tagged proteins in HEK-293 cells and analyzed their post-translational modification patterns by two-dimensional gel electrophoresis (2D-GE). We reasoned that if any cellular pool of Pfn1 is phosphorylated on T89, rendering it non-phosphorylatable should result in a basic charge shift of the isoelectric profile of Pfn1 in 2D GE. We observed multiple charged states of myc-Pfn1 with the second spot representing the predominant pool of myc-Pfn1. Rendering T89 non-phosphorylatable by alanine substitution resulted in basic shift of a small population of myc-Pfn1 (Fig 8). As spot #2 accounts for >95% of total pool of myc-Pfn1, we used the relative intensities of spot #2 at a very low exposure (that does not saturate the signal of this spot) between WT- and T89A-Pfn1 IEFs as a correction factor for the loading control for the two groups. Using this correction factor, we estimated that T89A substitution led to an approximately 8.9-fold (mean of two independent experiments) increase in the ratio of intensity of spot #1 to that of spot #3 (a measure of net basic charge shift). This finding is consistent with the scenario that a small fraction of cellular Pfn1 could be post-translationally modified on the T89 residue in cells. Note that without direct mass-spectrometry-based evidence of T89 phosphorylation, we could not absolutely rule out an alternative interpretation that T89A substitution may render a certain pool of Pfn1 resistant to phosphorylation or other types of post-translational modification on sites other than T89.

## Discussion

It is well recognized that Pfn1 is a vital cog in the machinery that regulates actin polymerization in cells. Therefore, how Pfn1’s interactions with actin and other major controllers of actin polymerization are regulated is a fundamentally important biological question in the context of actin-driven cellular events. There is emerging evidence that phosphorylation plays an important role in tuning Pfn1’s ligand interactions. At present, only two phosphorylation events (Y128, S137) have been directly linked to functional alterations of Pfn1. This study demonstrates that Pfn1 can be directly phosphorylated by PKA, and accordingly, post-translationally modified in a PKA-dependent manner in cells. We identified at least three PKA phosphorylation sites (S56/S57, T89, S91/T92). As phosphopeptides of Pfn1 involving all of these residues were previously found in various proteomics screens [10,19,20], these phosphorylations appear to be bona fide *in vivo* events. By MDS, we established similarities between the intramolecular changes caused by actual T89 phosphorylation and T89D substitution suggesting T89D-Pfn1 reasonably mimics a T89-phosphorylated state of Pfn1. We further showed that T89D-Pfn1 exhibits hallmarks of proteins in an alternative three-dimensional conformation including protein aggregation when expressed in cells, insolubility and marking for proteolytic degradation. Therefore, T89 appears to be a structurally critical residue of Pfn1 that is a target for phosphorylation leading to significant biochemical consequence.

Previous studies have mutated residues near and adjacent to T89, including R88 and K90, with no apparent impact on solubility [22,23]. Recently, several Pfn1 mutations (C71G, M114T, E117G and G118V) have been linked to pathogenesis of familial Amyotrophic Lateral Sclerosis (ALS) [24]. Except for E117G, the other three mutations were shown to result in protein aggregation and insolubility of Pfn1 in cells, very similar to the phenotypes exhibited by phosphomimetic mutation of the T89 residue demonstrated in the present study. Another phenotypic parallel between the ALS-linked mutations and the T89D mutation was the inhibitory effect on actin polymerization. ALS-linked mutations lie in close proximity to the actin-binding residues of Pfn1 while the backbone of the T89 residue directly interacts with Y166 of actin. As ALS-linked mutations still preserved a significant NP-40 soluble pool of Pfn1, a co-immunoprecipitation assay was feasible in that study to demonstrate that ALS-linked mutations result in reduced actin-binding of Pfn1. However, in our case, the insolubility issue of the T89D mutant was much more severe, precluding us from performing similar biochemical assays. *In silico* analyses predicted that phosphorylation of T89 could potentially result in an ‘actin-friendly’ conformation because the backbone oxygen of T89 is more accessible to Y166 of actin through destabilization of intramolecular T89:F98 interaction. T89D-Pfn1:actin co-clusters in cells even in the near absence of endogenous Pfn1 expression is consistent with a direct interaction of T89D-Pfn1 and actin, as predicted by the *in silico* analyses.

Is our *in silico* prediction of the increased strength of Pfn1:actin binding conferred by T89 phosphorylation consistent with reduced level of actin polymerization in cells upon T89D-Pfn1 overexpression? Pfn1 has dual effects on actin cytoskeleton. Pfn1 can either inhibit actin polymerization (by sequestering G-actin, inhibiting spontaneous or Arp2/3-mediated actin nucleation) or promote barbed-end directed actin polymerization. Even for barbed-end elongation, Pfn1 must also dissociate from G-actin following shuttling of G-actin to the barbed ends. If T89 phosphorylation dramatically enhances Pfn1’s affinity for actin to a point that dissociation of the Pfn1:actin complex is greatly inhibited, in an overexpression setting T89D-Pfn1 should not only competitively inhibit the endogenous Pfn1:actin interaction, but it could also act as a G-actin sequestering protein resulting in an overall inhibition of actin polymerization. There is, however, an alternative possibility that T89D-Pfn1 induces local actin polymerization at the sites of its aggregation and in turn make less G-actin available for actin polymerization elsewhere in the cell. We think this is unlikely since phalloidin staining did not reveal any F-actin clusters at the sites of T89D-Pfn1 aggregates. Although it remains to be seen whether activating the PKA pathway triggers T89 phosphorylation of Pfn1 in cells, if T89-phosphorylation of Pfn1 inhibits actin polymerization of Pfn1 as suggested by the F-actin phenotype of T89D-Pfn1 expressors in this study, it would be at least consistent with the recent finding of FSK-induced stellation of astrocytes in a Pfn-dependent manner [18].

Another interesting finding of this study was the faster protein turnover of Pfn1 conferred by the phosphomimetic mutation on the T89 residue. Although the structural basis for this observation is currently unclear, one simple explanation could be that T89D substitution affects protein folding of Pfn1, and that the misfolded protein becomes a target for degradation. Previous proteomic studies have found evidence of ubiquitination of the adjacent residue K90 [25,26]. Thus, it is possible that T89 phosphorylation induces a large structural change through destabilizing the beta sheet structure which increases the accessibility of K90 (or other ubiquitylable lysine residues) to ubiquitin-ligases and priming Pfn1 for degradation. If this is true, T89 phosphorylation could be a triggering event for local protein turnover of Pfn1.

In conclusion, we have identified T89 phosphorylation as a novel post-translational modification of Pfn1 which has important biochemical consequence on Pfn1. The actual biological significance of T89 phosphorylation still needs to be resolved in future studies. Although phosphorylation of other PKA sites identified herein (S57, S91/T92) residues did not show any obvious change in Pfn1’s ability to co-precipitate actin and PLP ligand in a GST-pull down assay, without a quantitative binding assay, we cannot absolutely rule out subtle changes in ligand affinity of Pfn1 due to these mutations. Furthermore, whether phosphorylation of any these residues has any influence on other post-translational modifications or impacts other functionalities of Pfn1 (such as PPI binding), particularly if they occur simultaneously, need further investigation. Future studies should also examine whether PKA signaling influences actin dynamics and actin-dependent biological processes such as cell migration through phosphorylation of Pfn1.

## Supporting Information

S1 Fig HEK-293 cells expressing myc-tagged T89D-Pfn1were lysed with either non-denaturing (containing 1% NP-40) or denaturing (containing 1% NP-40, 2% SDS for one buffer and the other with 6M urea in addition) extraction buffers. Cell lysates were immunoblotted with anti-myc antibody to demonstrate that myc-tagged T89D-Pfn1 is insoluble in non-denaturing lysis buffer.
(TIF)

S2 Fig Quantitative RT-PCR shows comparable mRNA levels of all three GFP-tagged Pfn1 constructs in HEK-293 cells. (TIF)

S3 Fig Actin and tubulin (loading control) immunoblots of extracts prepared from MDA-231 cells expressing the indicated Pfn1 constructs as EGFP-tagged proteins. (TIF)

S1 Table Primer sequences used for site-directed mutagenesis of Pfn1. (DOC)

S2 Table Phosphorylation sites and associated kinases of human and mouse Pfn1, as predicted by NetphosK (threshold set at the default value of 0.5) and KinasePhos (prediction accuracy set at 0.90). In addition to predicted sites that are annotated by the plus sign, this table also includes a few kinase-specific experimentally validated phosphorylations, e.g. Src/Y128, PKC/S137 and ROCK/S137, even though these did not meet the cut-off or prediction accuracy criteria set in our analyses.
(TIF)

## References

[1] Bugyi B, Carlier MF (2010) Control of actin filament treadmilling in cell motility. Annu Rev Biophys 39: 449–470. 10.1146/annurev-biophys-051309-103849 20192778

[2] Ding Z, Bae YH, Roy P (2012) Molecular insights on context-specific role of profilin-1 in cell migration. Cell Adh Migr 6: 442–449. 10.4161/cam.21832 23076048 PMC3496682

[3] Geese M, Loureiro JJ, Bear JE, Wehland J, Gertler FB, et al (2002) Contribution of Ena/VASP proteins to intracellular motility of Listeria requires phosphorylation and proline-rich core but non F-actin binding or multimerization. Molecular Biology of the Cell 13: 2383–2396. 12134077 10.1091/mbc.E02-01-0058 PMC117321

[4] Hansen SD, Mullins RD (2010) VASP is a processive actin polymerase that requires monomeric actin for barbed end association. J Cell Biol 191: 571–584. 10.1083/jcb.201003014 21041447 PMC3003327

[5] Suetsugu S, miki H, Takenawa T (1998) The essential role of profilin in the assembly of actin for microspike formation. EMBO Journal 17: 6516–6526. 9822597 10.1093/emboj/17.22.6516 PMC1170999

[6] Suetsugu S, Miki H, Takenawa T (1999) Distinct roles of profilin in cell morphological changes: mircospikes, membrane ruffles, stress fibers, and cytokinesis. FEBS Letters 457: 470–474. 10471831 10.1016/s0014-5793(99)01086-8

[7] Lassing I, Lindberg U (1985) Specific interaction between phosphatidylinositol 4,5-bisphosphate and profilactin. Nature 314: 472–474. 2984579 10.1038/314472a0

[8] Kasina S, Rizwani W, Radhika KV, Singh SS (2005) Nitration of profilin effects its interaction with poly (L-proline) and actin. J Biochem 138: 687–695. 16428297 10.1093/jb/mvi163

[9] Kasina S, Wasia R, Fasim A, Radhika KV, Singh SS (2006) Phorbol ester mediated activation of inducible nitric oxide synthase results in platelet profilin nitration. Nitric Oxide 14: 65–71. 16288977 10.1016/j.niox.2005.09.008

[10] Fan Y, Arif A, Gong Y, Jia J, Eswarappa SM, et al (2012) Stimulus-dependent phosphorylation of profilin-1 in angiogenesis. Nat Cell Biol 14: 1046–1056. 10.1038/ncb2580 23000962 PMC3619429

[11] Shao J, Welch WJ, Diprospero NA, Diamond MI (2008) Phosphorylation of profilin by ROCK1 regulates polyglutamine aggregation. Mol Cell Biol 28: 5196–5208. 10.1128/MCB.00079-08 18573880 PMC2519718

[12] Satish K, Padma B, Munugalavadla V, Bhargavi V, Radhika K, et al (2004) Phosphorylation of profilin regulates its interaction with actin and poly (L-proline). Cell Signalling 16: 589–596. 14751544 10.1016/j.cellsig.2003.10.001

[13] Ding Z, Lambrechts A, Parepally M, Roy P (2006) Silencing profilin-1 inhibits endothelial cell proliferation, migration and cord morphogenesis. J Cell Sci 119: 4127–4137. 16968742 10.1242/jcs.03178

[14] Shevchenko A, Tomas H, Havlis J, Olsen JV, Mann M (2006) In-gel digestion for mass spectrometric characterization of proteins and proteomes. Nat Protoc 1: 2856–2860. 17406544 10.1038/nprot.2006.468

[15] Case DA, Babin V, Berryman JT, Betz RM, Cai Q, et al (2014) AMBER 14. University of California, San Francisco.

[16] Craft JW Jr, Legge GB (2005) An AMBER/DYANA/MOLMOL phosphorylated amino acid library set and incorporation into NMR structure calculations. J Biomol NMR 33: 15–24. 16222554 10.1007/s10858-005-1199-0

[17] Michaud-Agrawal N, Denning EJ, Woolf TB, Beckstein O (2011) MDAnalysis: A toolkit for the analysis of molecular dynamics simulations. J Comput Chem 32: 2319–2327. 10.1002/jcc.21787 21500218 PMC3144279

[18] Schweinhuber SK, Messerschmidt T, Hansch R, Korte M, Rothkegel M (2015) Profilin isoforms modulate astrocytic morphology and the motility of astrocytic processes. PLoS One 10: e0117244 10.1371/journal.pone.0117244 25629407 PMC4309604

[19] Hornbeck PV, Kornhauser JM, Tkachev S, Zhang B, Skrzypek E, et al (2012) PhosphoSitePlus: a comprehensive resource for investigating the structure and function of experimentally determined post-translational modifications in man and mouse. Nucleic Acids Res 40: D261–270. 10.1093/nar/gkr1122 22135298 PMC3245126

[20] Olsen JV, Vermeulen M, Santamaria A, Kumar C, Miller ML, et al (2010) Quantitative phosphoproteomics reveals widespread full phosphorylation site occupancy during mitosis. Sci Signal 3: ra3 10.1126/scisignal.2000475 20068231

[21] Korupolu RV, Achary MS, Aneesa F, Sathish K, Wasia R, et al (2009) Profilin oligomerization and its effect on poly (L-proline) binding and phosphorylation. Int J Biol Macromol 45: 265–273. 10.1016/j.ijbiomac.2009.06.001 19523483

[22] Bjorkegren C, Rozycki M, Schutt CE, Lindberg U, Karlsson R (1993) Mutagenesis of human profilin locates its poly(L-proline)-binding site to a hydrophobic patch of aromatic amino acids. FEBS Lett 333: 123–126. 8224149 10.1016/0014-5793(93)80388-b

[23] Skare P, Karlsson R (2002) Evidence for two interaction regions for phosphatidylinositol(4,5)-bisphosphate on mammalian profilin I. FEBS Lett 522: 119–124. 12095630 10.1016/s0014-5793(02)02913-7

[24] Wu CH, Fallini C, Ticozzi N, Keagle PJ, Sapp PC, et al (2012) Mutations in the profilin 1 gene cause familial amyotrophic lateral sclerosis. Nature 488: 499–503. 10.1038/nature11280 22801503 PMC3575525

[25] Kim W, Bennett EJ, Huttlin EL, Guo A, Li J, et al (2011) Systematic and quantitative assessment of the ubiquitin-modified proteome. Mol Cell 44: 325–340. 10.1016/j.molcel.2011.08.025 21906983 PMC3200427

[26] Wagner SA, Beli P, Weinert BT, Nielsen ML, Cox J, et al (2011) A proteome-wide, quantitative survey of in vivo ubiquitylation sites reveals widespread regulatory roles. Mol Cell Proteomics 10: M111 013284. 10.1074/mcp.M111.013284 PMC3205876 21890473

