# Supplementary figures and images for: Threonine 89 Is an Important Residue of Profilin-1 That Is Phosphorylatable by Protein Kinase A
Source: PLoS One. 2016 May 26;11(5):e0156313. doi: 10.1371/journal.pone.0156313 (PMC4882052; doi:10.1371/journal.pone.0156313)

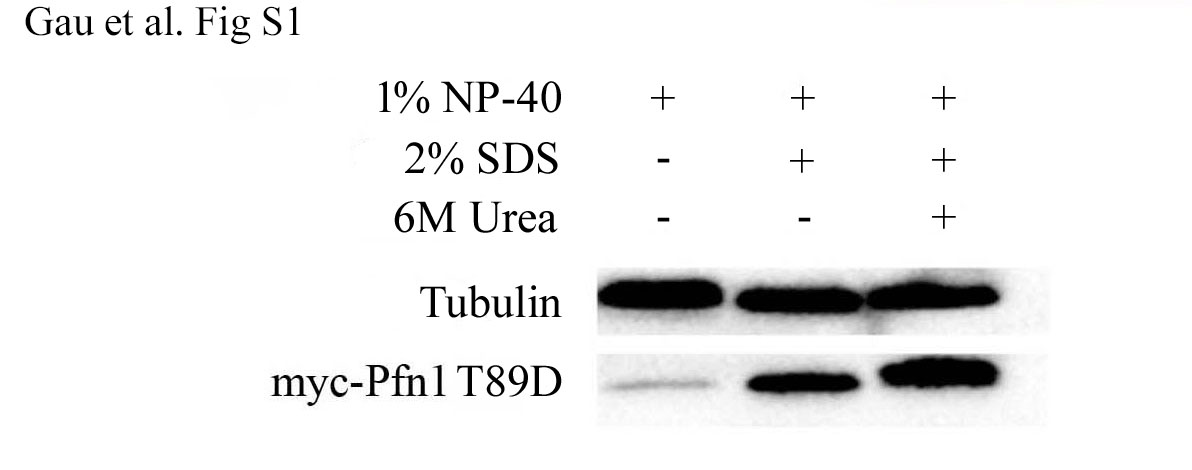

Supplement: S1 Fig — Cell lysates were immunoblotted with anti-myc antibody to demonstrate that myc-tagged T89D-Pfn1 is insoluble in non-denaturing lysis buffer. (TIF) [file pone.0156313.s001.tif]

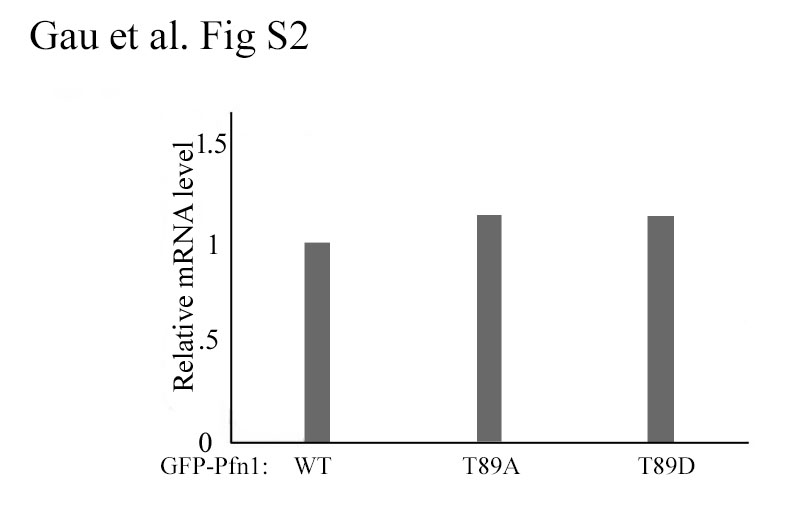

Supplement: S2 Fig — (TIF) [file pone.0156313.s002.tif]

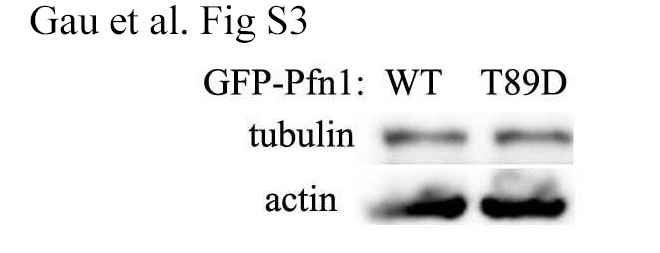

Supplement: S3 Fig — (TIF) [file pone.0156313.s003.tif]

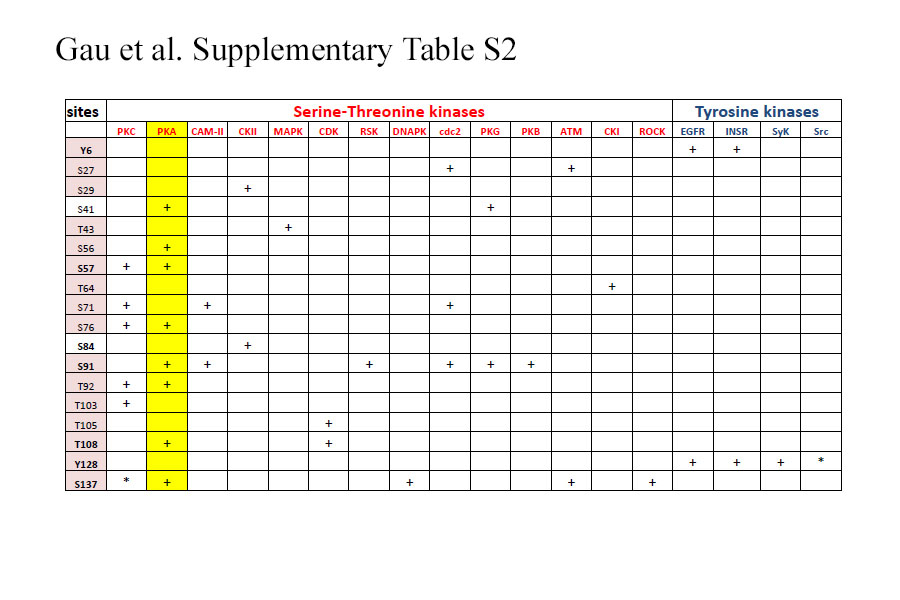

Supplement: S2 Table — In addition to predicted sites that are annotated by the plus sign, this table also includes a few kinase-specific experimentally validated phosphorylations, e.g. Src/Y128, PKC/S137 and ROCK/S137, even though these did not meet the cut-off or prediction accuracy criteria set in our analyses. (TIF) [file pone.0156313.s005.tif]
