# Supplementary material for: Threonine 89 Is an Important Residue of Profilin-1 That Is Phosphorylatable by Protein Kinase A
Source: PLoS One. 2016 May 26;11(5):e0156313. doi: 10.1371/journal.pone.0156313 (PMC4882052; doi:10.1371/journal.pone.0156313)
Supplement: S1 Table — (DOC) [file pone.0156313.s004.doc]

**Gau et al. Supplementary Table S1**

| **Mutation** | **Primer Sequence** |
| --- | --- |
| **S57D**  **T89D**  **S91D**  **T92D**  **T89A** | sense: 5’- GGCAAAGACCGGTCAGATTTTTTCGTC-3’  sense: 5’- GGATCTTCGTGACAAGAGCACCGG-3’  sense: 5’- CGTACCAAGGACACCGGAGGAG-3’  sense: 5’-CCAAGAGCGACGGAGGAGCC-3’  sense: 5’- GGACTTCGTGCCAAGAGCACCGG-3’ |
